# Convalescing Cluster Configuration Using a Superlative Framework

**DOI:** 10.1155/2015/180749

**Published:** 2015-10-12

**Authors:** R. Sabitha, S. Karthik

**Affiliations:** ^1^Department of Information Technology, Info Institute of Engineering, Coimbatore 641107, India; ^2^Department of CSE, SNS College of Technology, Coimbatore 641035, India

## Abstract

Competent data mining methods are vital to discover knowledge from databases which are built as a result of enormous growth of data. Various techniques of data mining are applied to obtain knowledge from these databases. Data clustering is one such descriptive data mining technique which guides in partitioning data objects into disjoint segments. *K*-means algorithm is a versatile algorithm among the various approaches used in data clustering. The algorithm and its diverse adaptation methods suffer certain problems in their performance. To overcome these issues a superlative algorithm has been proposed in this paper to perform data clustering. The specific feature of the proposed algorithm is discretizing the dataset, thereby improving the accuracy of clustering, and also adopting the binary search initialization method to generate cluster centroids. The generated centroids are fed as input to *K*-means approach which iteratively segments the data objects into respective clusters. The clustered results are measured for accuracy and validity. Experiments conducted by testing the approach on datasets from the UC Irvine Machine Learning Repository evidently show that the accuracy and validity measure is higher than the other two approaches, namely, simple *K*-means and Binary Search method. Thus, the proposed approach proves that discretization process will improve the efficacy of descriptive data mining tasks.

## 1. Introduction

Conventional database analysis techniques are not practically good at extracting knowledge from enormous databases. Proficient data mining methods are vital to discover knowledge from these databases. Effective techniques in data mining techniques steer in finding out useful acquaintances from raw data [1–5]. Data clustering is one such technique which guides in partitioning data objects into disjoint segments. Market segmentation, image processing, and bioinformatics are popular amongst the applications of data clustering [6, 7]. Numerous algorithms are available in the literature to direct the clustering process. These algorithms have specialised features that make clustering possible in diverse ways. One among these algorithms is the versatile *K*-means algorithm which is simple, robust, and easy to employ [8]. It is an iterative-partitioning algorithm which partitions the data into *K* clusters, where *K* is a user specified parameter. It starts with *K* initial centroids and iteratively performs two steps: assigning the data object to the cluster whose centroid is the nearest to the object and updating the clusters' centroid [9]. The major objectives of clustering are to satisfy and to maintain suitable distance measures using Euclidean distance or Manhattan distance [10]. In spite of its simplicity and ease *K*-means has few disadvantages. The outcome of *K*-means segmentation is influenced by initial centroid selection and hence the partitions produced force the outcome to be trapped in local optima [11].

Many adaptations in the form of heuristic approaches are made to the *K*-means algorithm which makes it more flexible and robust. The approaches are, namely, Simulated Annealing, Ant Colony Optimization (ACO), Tabu Search, Genetic Algorithm, Optimization approach using Honey-Bee Mating, Particle Swarm Optimization (PSO), hybrid technique based on *K*-means, ACO, and PSO, Big Bang-Big Crunch, Artificial Bee Colony, Gravitational Search algorithm, and Binary Search algorithm [12–22]. Though these heuristic approaches enhance the efficacy of *K*-means clustering, they endure several drawbacks like complication in their structure and implementation, limited eminence in their results, optimization problems, and result convergence problems [23]. The limited eminence in the results leads to less accuracy. To overcome these limitations and to achieve accurate results in descriptive data mining tasks a superlative framework is proposed in this paper which clusters the data objects with high efficacy. The major perspective of this proposed method is to enhance the accuracy of the data clustering process.

## 2. Related Work

Various algorithms are available in the literature to guide in the clustering process. These algorithms have some dedicated features that make clustering possible in diverse ways. One among these algorithms is the versatile *K*-means algorithm which is simple, robust, and easy to employ [24]. Many adaptations in the form of heuristic approaches are made to this *K*-means algorithm which makes it more flexible and robust.

Diverse centroid initializations produce dissimilar clustering results since *K*-means clustering algorithm tends to local minima. To conquer local minima the algorithm can be executed various times with numerous dissimilar initial centroids for a given *K* and then deciding the clusters with the nominal squared error. No global and competent way exists for generating the preliminary partitions. The final cluster points differ for various trials from the diverse preliminary centroids.

Peña, Larrañaga, and Lozano measured the efficiency, convergence speed criteria, effectiveness, and robustness of random initialization with other initialization techniques proposed by Kaufman and Rousseeuw [6]. The experimental results proved that the random method and Kaufman's method perform much better than the others in terms of efficiency, effectiveness, and robustness. Further measuring the convergence speed, the authors suggested Kaufman's technique to be the efficient one.

Bradley and Fayyad proposed an enhancement that initially executes *K*-means method *R* times to obtain *R* accidental partitions from the input dataset [25]. The results obtained by blending the solution belonging to the *R* clusters are reclustered *R* times, using the subset solution as an initial guess. The preliminary centroids for the entire dataset are finalized by selecting the ones with nominal error.

Likas et al. developed a universal method involving a series of segmenting trials with the size of clusters ranging from 1 to *k* [26]. The preceding *k* − 1 points are set and the fresh points are chosen by investigating the entire base. The algorithm proved efficient and was independent of the initial partitions. The computational complexity becomes the drawback of the algorithm since the algorithm executes *n* number of trials for all *k* values. The repetitive procedure thus does not assure result convergence.

Krishna and Murty added novel methods in their amalgam scheme to attain speedy convergence and global solutions [27]. They designed the enhancement based on the variance between two data points, thus making it stay away from being trapped in confined optima.

*K*-means with an adaptive learning strategy is illustrated by Chinrungrueng and Séquin [28]. It can be tuned without concerning any user activities and is solely dependent on the within-group variations.

Patanè and Russo projected an enhanced technique [29], using a roulette method involving genetic algorithms which is nonsusceptible to centroid spawning problems.

Tzortzis and Likas implemented MinMax algorithm [9], a method that eliminates centroid spawning problem by varying its purpose. The algorithm starts from arbitrarily selected centroids and maintains a maximum value of intraclass distance rather than the summation of the intraclass distances. Exclusively, a value is related to each segment; that is, segments having higher variations are allotted high values; thus a weighted edition is achieved. The projected method restricts generation of huge variation clusters and produces efficient results, in spite of the initialization process. Rather this methodology employs a factor *Q* which tunes towards disciplining its cluster generation. The algorithm requires this parameter to be specified prior to execution which is considered as a drawback.

Alsultan and Selim [12] proposed Simulated Annealing (SA) approach where the segmentation problem congregates to a global solution.

Kim and Ahn [13] used Genetic Algorithm (GA) which was effective on NP-complete global optimization problems and provided good near-optimal solutions in reasonable time.

Al-Sultan [14] adapted Tabu Search which incorporates metaheuristic approach and was superior over local search clustering algorithms.

Fathian et al. [15] proposed Optimization using Honey-Bee Mating (HBMO) which incorporates optimization using swarm-based approach.

Shelokar et al. [16] implemented Ant Colony Optimization (ACO) which uses distributed agents which imitate the way ants find a minimal path from their home to food source.

Chen and Ye [17] used Particle Swarm Optimization (PSO) which searches for the cluster center in the arbitrary data set automatically.

Niknam and Amiri [18] projected an amalgam method based on *K*-means using both ACO and PSO which deciphers nonlinear clustering problem using an evolutionary approach.

Hatamlou et al. [19] incorporated Big Bang-Big Crunch technique based on one of the theories of the evolution of the universe.

Karaboga and Ozturk [20] implemented Artificial Bee Colony (ABC) which modeled the clever foraging action of a honey bee flock and was competently employed to perform multivariate clustering.

Hatamlou et al. [21] used Gravitational Search approach which helped the *k*-means algorithm to not only escape from local optima but also increase the convergence speed of the algorithm.

Hatamlou [22] developed a Binary Search algorithm to discover superior clusters and the methodology converged to identical result in diverse runs.

## 3. Proposed Methodology

The proposed method is a segmentation based method that receives *k*—the size of segments—as input and partitions the dataset into *k* clusters. It is a simple and superlative method which first discretizes the dataset, calculates preliminary centroids, and then allocates each and every object in the input base to the closest centroids. Hence the framework clusters the data objects with high efficacy.

The methodology involves discretization techniques [30] which transforms continuous data into discrete ones. The dataset *D* = {*d*_1_, *d*_2_,…, *d*_*n*_} with “*f*” continuous attributes is transformed into discrete values for attributes followed by identifying the initial centroids *C*_1_, *C*_2_,…, *C*_*k*_, given *K* the number of clusters to be generated. These centroids are used by the *K*-means data clustering approach to segment the data objects of *D* into exactly *K* clusters given by *C* = {*C*_1_, *C*_2_,…, *C*_*k*_}, thus maximizing accuracy.

The main objective of this proposed approach is (1) to adapt simple structures in representation, (2) to develop a methodology which is effortless and easy to implement, (3) to provide robust and trustworthy approach, (4) to produce accurate clusters, and (5) to generate clusters quickly.

Contributions of this work are as follows:

(i) *Proposing a Framework to Cluster the Input Dataset*. A superlative framework is proposed with three phases described in Sections 3.1, 3.2, and 3.3.
(ii) *Concrete Description of Typical Discretization Process*. Discretization phase converts the continuous valued features into discrete values which are further quantile binned. As a result of the discretization and binning process, reformed data objects are obtained.
(iii) *Adaptation of Binary Search Method*. Binary Search method is adapted to spawn the preliminary centroids, where the dataset is split into equal parts based on the number of clusters required. Then split point* S* is found which is used to spawn the initial centroids.
(iv) *Modified K-Means Approach*. The algorithm *K*-means employs the centroids generated from the previous step (which is not the case in general *K*-means) as initial points and assigns the data points to the nearest centroids, followed by recomputation of cluster centroids.
(v) *Algorithmic Representation of the Phases in Framework*. The algorithms for the three phases are depicted in Algorithms 1, 2, and 3.
(vi) *Demonstration of Applying the Framework on Benchmark Datasets*. Performance measurement and effectiveness evaluation of the proposed methodology on benchmarked datasets are done and results are shown in Tables 1 and 2 and discussed in Sections 4.1 and 4.2.
(vii) *Comparative Analysis of the Proposed Approach with K-Means and Binary Search Method*. For the comparative study, simple *K*-means and Binary Search methods are considered. To demonstrate the strength of the proposed approach the former mentioned methods are compared with the latter one. The metrics are deliberated and sketched for various datasets. The results are shown in Tables 3 and 4 and discussed in Section 4.3.
(viii) *Comprehensive Assessment of Comparative Results*. The efficacy of the proposed methodology is discussed in Section 4.4.

The major phases of the proposed framework are as follows:

 Phase I: discretization,
 Phase II: identifying initial centroids,
 Phase III: *K*-means clustering.

**Figure 1:**
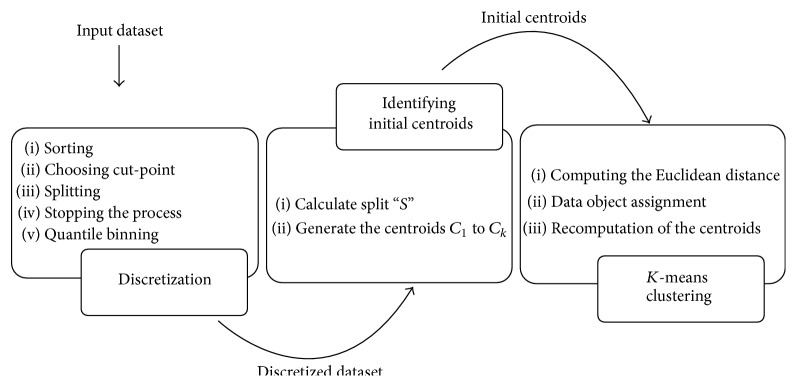
Proposed framework.

Discretization phase converts the continuous valued features into discrete values which are further quantile-binned. As a result of the discretization and binning process, reformed data objects are obtained. Generally *K*-means algorithm generates its initial centroids randomly. Various initial centroid generation methods have been developed to improve the process of *K*-means clustering. The proposed framework uses a novel Binary Search method to generate the initial *K* cluster centroids. Upon generation, the data objects of *D* are allotted to the closest cluster and the respective cluster centroids are recomputed. This process iterates until all the data objects are assigned to their corresponding nearest cluster. The data clustering model of the proposed framework is shown in Figure 1.

### 3.1. Phase I: Discretization

Data usually may be in a mixed format; it may be discrete, nominal, or continuous. Discrete data are ordinal; that is, they possess some order amongst them. The number of values in discrete type is few or finite which makes it easy in learning. Discrete features are easy to understand, use, and explain. It makes learning faster and accurate. Discretization process may be carried out in various ways based on the type of data and usage [30].

**Figure 2:**
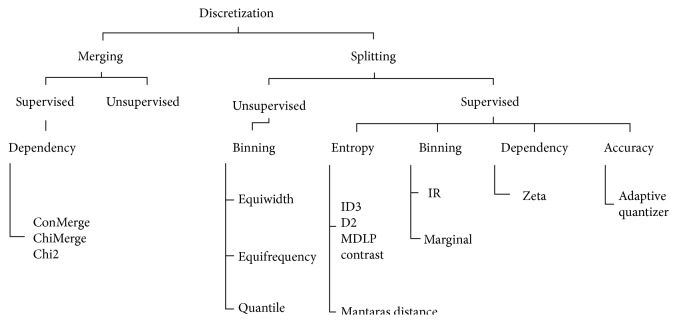
Discretization framework.

Discretization can be categorized as local or global, static or dynamic, top-down or bottom-up, direct or incremental, and supervised or unsupervised. The discretization framework is depicted in Figure 2.

**Figure 3:**
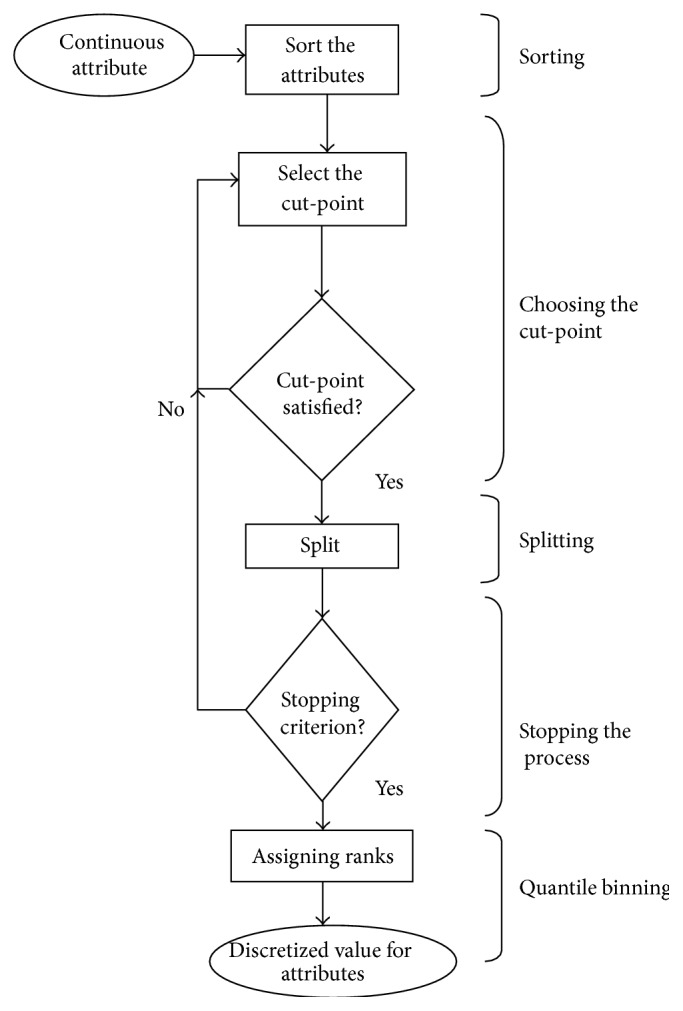
Process of discretization.

The steps of the discretization process are given in Algorithm 1. The process involves sorting: arranging the continuous valued features *f* of *D* either in ascending or in descending order (step (3)); choosing the cut-points “cp”: finding the best “split-point” to divide a range of continuous values (steps (4) to (6)); splitting: evaluating the best cut-point “cp” and splitting the range of continuous values (steps (5) and (7)). Evaluation involves checking for simplicity, accuracy, and consistency; stopping the process: controlling the overall discretization process based on the “arity” (number of intervals or partitions) (steps (3) to (7) repeated iteratively); quantile binning: assigning ranks to the features based on the quantile to which the object belongs (step (8)). The process is shown in Figure 3 [30].

### 3.2. Phase II: Identifying Initial Centroids

The identification of initial centroids is tailored from the Binary Search algorithm. The dataset is split into equal parts based on the cluster size *K*. The split point of the partitions is represented by *S* and is calculated by using the formula

(1) $$\begin{array}{c} S=\frac{\max\left(D\right)-\min\left(D\right)}{k}, \end{array}$$

where max⁡(*D*) and min⁡(*D*) correspond to the upper and lower limit values of the data objects in *D* with reference to the whole dataset [22].

The split *S* is used to spawn the initial centroids. The centroid of the clusters *C*_1_, *C*_2_,…, *C*_*k*_ is generated using

(2) $$\begin{array}{c} C_{i}=\min\left(\;D\;\right)+\left(\;i-1\;\right)*S,\;i=1,2,\ldots ,k. \end{array}$$

The process of identifying initial centroids by adapting Binary Search method is given in Algorithm 2.

### 3.3. Phase III: *K*-Means Clustering

The *K*-means approach segments the data objects of *D* into *K* clusters.  Algorithm 3 illustrates the algorithm. In the proposed methodology *K*-means algorithm starts off with assigning *K* initial centroids identified in Phase II (which is not the case in general *K*-means) to *K* clusters and repeatedly performs the following steps:

(i) Compute the Euclidean distance.
(ii) Assign the data objects in *D* to their corresponding clusters depending on the Euclidean distance.
(iii) Recompute/revise the cluster centroids [31].

The Euclidean distance, one of the superlative measures to be used in *K*-means clustering algorithm, computes the distance between two data objects [32, 33]. The distance between two data objects *d*_*i*_ and *d*_*j*_ using this measure is given by

(3) $$\begin{array}{c} \text{Dist}\left(\;d_{i},d_{j}\;\right)=\sqrt{\overset{m}{\underset{l=1}{\sum }}{\left(d_{i}^{l}-d_{j}^{l}\right)}^{2}}. \end{array}$$

The major perspective of this proposed method is to enhance the efficacy of the data clustering process. The efficacy of clustering the input dataset *D* with *n* data objects into *C* = {*C*_1_, *C*_2_,…, *C*_*k*_} is measured as follows:

(4) $$\begin{array}{c} \text{Accuracy}=\frac{\sum_{i=1}^{i=k}C_{i}}{n}. \end{array}$$

In addition to accuracy, the validity of the data clustering process is evaluated using* Davies-Bouldin index* (DB index) [34] given by

(5) $$\begin{array}{c} DB=\frac{1}{k}\overset{k}{\underset{i=1}{\sum }}\underset{j\neq i}{\max}\left(\;\frac{\sigma_{i}+\sigma_{j}}{dist\left(c_{i},c_{j}\right)}\;\right), \end{array}$$

where *σ*_*i*_ represents the calculated middling distance of every data object in cluster to its corresponding centroid, *k* represents the count of groups/clusters to be formed, centroid of any cluster *x* is represented by *c*_*x*_, and dist(*c*_*i*_, *c*_*j*_) measures the Euclidean distance between centroids *c*_*i*_ and *c*_*j*_ [24, 35].

## 4. Experimentation and Discussions

This segment elaborates the outcome of the proposed methodology and its effectiveness in terms of accuracy and DB index. The scheme is tested on datasets like Iris, Wine, Cancer, and Vowel obtained from the repository of databases, UC Irvine Machine Learning Repository [36]. This section is subcategorized into performance measurement, effectiveness evaluation, comparative analysis, and discussions. Performance measurement analyses the accuracy, effectiveness evaluation measures the validity using DB index, and comparative analysis does a comprehensive assessment of comparative results.

### 4.1. Performance Measurement

**Figure 4:**
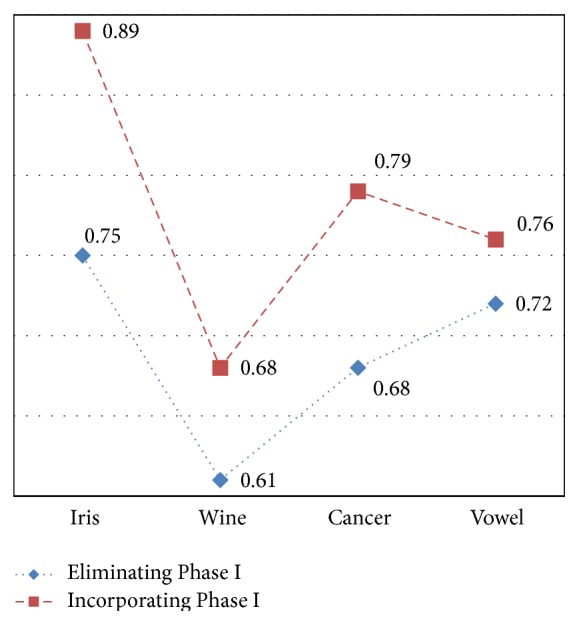
Accuracy of proposed method with and without Phase I.

The effectiveness of the planned methodology is measured based on accuracy using (4). The accuracy achieved for various datasets with and without using Phase I (discretization) is given in Table 1.  Figure 4 plots the accuracy of the methodology for various datasets. It is observed that the accuracy of the approach without using Phase I is 0.75, 0.61, 0.68, and 0.72 when applied to Iris, Wine, Cancer, and Vowel datasets which is probably less when compared to incorporating Phase I in the clustering process. The accuracy obtained after incorporating Phase I is 0.89, 0.68, 0.79, and 0.76, respectively. This proves the efficacy of discretization in improving the accuracy of clustering.

### 4.2. Effectiveness Evaluation

**Figure 5:**
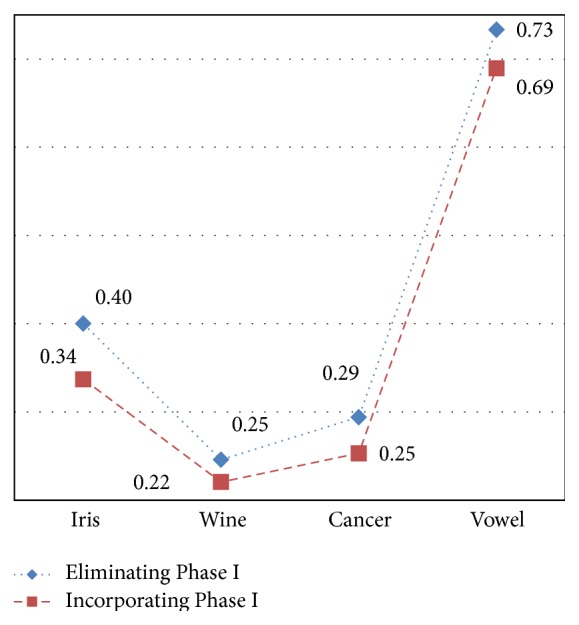
Validity of proposed method with and without Phase I.

Despite the fact that discretization improves accuracy which is evident from the above analysis, it is essential to evaluate the effectiveness of the clustering method in terms of validity. The validation measure DB index is deliberated using (5). The achieved values of DB index for various datasets are specified in Table 2.  Figure 5 sketches the validity measure for various datasets.

It is observed that the validity measure of the approach without using Phase I is high when compared to incorporating Phase I in the clustering process. The higher is the value of DB index the lower is its validity; hence incorporating Phase I is essential to improvise the validity of clusters. The values fall from 0.40 to 0.34, 0.25 to 0.22, 0.29 to 0.25, and 0.73 to 0.69 for Iris, Wine, Cancer, and Vowel datasets.

### 4.3. Comparative Analysis

For the comparative study, simple *K*-means and Binary Search method are considered. To demonstrate the strength of the proposed approach the former mentioned methods are compared with the latter one. The metrics are deliberated and sketched for various datasets.

It is noticed that the accuracy for the proposed methodology when applied to Iris dataset is 0.89, is 0.68 to Wine dataset, is 0.79 to Cancer dataset, and is 0.76 to Vowel dataset. The accuracy values of the simple *K*-means when applied to various datasets are 0.69, 0.58, 0.6, and 0.65. For Binary Search method the values obtained are 0.75, 0.61, 0.68, and 0.72, respectively. The accuracy values of the proposed approach are significantly high when compared to the other two approaches.

**Figure 6:**
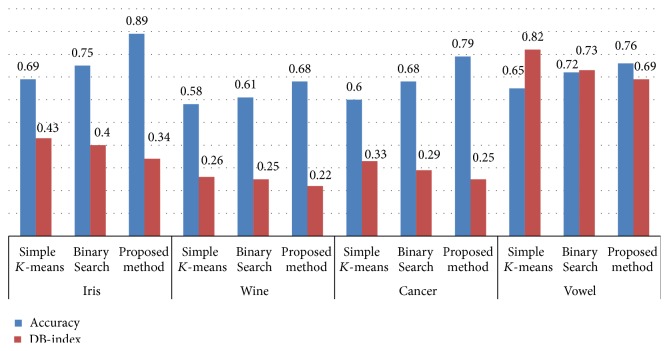
Comparative analysis of the algorithms based on accuracy and DB index.

Similarly the lower the DB index the higher the efficacy; the proposed algorithm achieves lower values of 0.34, 0.22, 0.25, and 0.69 when applied to Iris, Wine, Cancer, and Vowel datasets. For simple *K*-means the values are 0.43, 0.26, 0.33, and 0.82 and for Binary Search method the values are 0.4, 0.25, 0.29, and 0.73. The DB index values of the proposed approach are significantly low when compared to the other two approaches. The results are shown in Table 3 and Figure 6.

### 4.4. Discussions

The achievements of the objectives are portrayed in Table 4 which also discusses the rationale behind the objective contentment. The level of contentment is “high” for objectives 1 to 4 listed in Section 3. This is achieved due to the employment of efficient phases in the proposed approach. The contentment level is “medium” for responsiveness because of the discretization process.

Nevertheless the accuracy and validity measure of proposed method is efficient; it is obvious that the execution time is considerably high due to the discretization process. The clustering accuracy is highly important compared to the execution time and it is deliberately proved in the previous sections. The validity of the results obtained is fine for which the execution time can be compromised. In the future the focus will be on developing quickly responsive models.

## 5. Conclusion

A superlative framework has been proposed in this paper to perform data clustering. A particular feature of the approach is that it discretizes the dataset so as to improve the accuracy of clustering and also adapts the Binary Search initialization method to generate cluster centroids. These generated centroids are fed as input to* Phase III* which iteratively segments the data objects into respective clusters. The clustered results are measured for accuracy and validity. Experiments conducted by testing the approach on datasets from the UC Irvine Machine Learning Repository evidently show that the accuracy and validity measure is higher than the other two approaches, namely, simple *K*-means and Binary Search method. Thus the approach proves that discretization process will improve the efficacy of descriptive data mining tasks. Future work will focus on examining and developing methods which are quick and responsive.

## Figures and Tables

**Algorithm 1:**
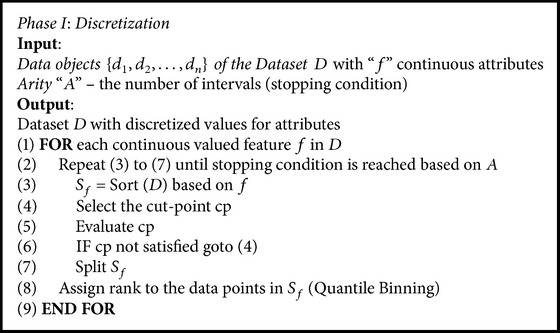
Steps in discretization.

**Algorithm 2:**
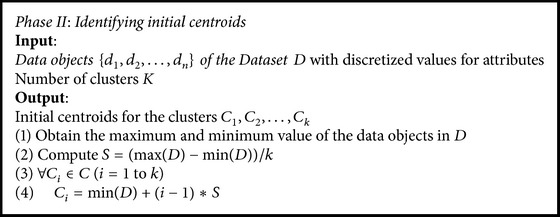
Identifying initial centroids.

**Algorithm 3:**
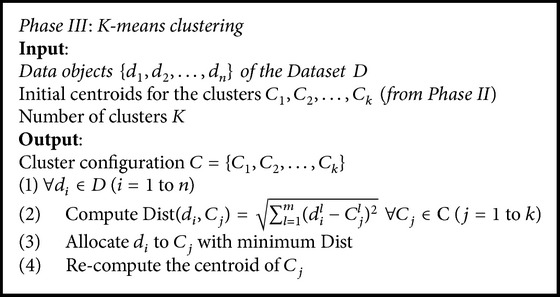
*K*-means clustering.

**Table 1:** Accuracy of proposed method.

Dataset	Proposed approach	
	Eliminating Phase I	Incorporating Phase I
Iris	0.75	**0.89**
Wine	0.61	**0.68**
Cancer	0.68	**0.79**
Vowel	0.72	**0.76**

**Table 2:** Validity of proposed method.

Dataset	Proposed approach	
	Eliminating Phase I	Incorporating Phase I
Iris	0.40	**0.34**
Wine	0.25	**0.22**
Cancer	0.29	**0.25**
Vowel	0.73	**0.69**

**Table 3:** Comparative analysis of the algorithms based on accuracy and DB index.

Dataset	Method	Accuracy	DB index
Iris	Simple *K*-means	0.69	0.43
	Binary Search	0.75	0.4
	Proposed method	**0.89**	**0.34**

Wine	Simple *K*-means	0.58	0.26
	Binary Search	0.61	0.25
	Proposed method	**0.68**	**0.22**

Cancer	Simple *K*-means	0.6	0.33
	Binary Search	0.68	0.29
	Proposed method	**0.79**	**0.25**

Vowel	Simple *K*-means	0.65	0.82
	Binary Search	0.72	0.73
	Proposed method	**0.76**	**0.69**

**Table 4:** Objective contentment.

Objectives	Contentment level	Rationale
To adapt simple structures in representation	High	Simple yet powerful phases in the framework
To develop a methodology which is effortless and easy to implement	High	
To provide robust and trustworthy approach	High	
To produce accurate clusters	High	

To generate clusters quickly	Medium	Execution time is high due to discretization process
